# Supplementary material for: Proteoform identification using multiplexed top-down mass spectra
Source: bioRxiv. 2025 Feb 8:2025.02.05.636727. Preprint. [Version 1] doi: 10.1101/2025.02.05.636727 (PMC11839095; doi:10.1101/2025.02.05.636727)
Supplement: Supplement 1 [file media-1.pdf]

Proteoform identification using multiplexed top-down mass spectra

(Supplemental materials)

Zhige Wang[1], Xingzhao Xiong[2], and Xiaowen Liu[2]

[1]Department of Computer Science, Tulane University, New Orleans, Louisiana, 70112, United States

[2]Deming Department of Medicine, Tulane University, New Orleans, Louisiana, 70112, United States

Tables

Table S1: Parameter settings of TopFD

Input Parameter	Value
Maximum charge	30
Maximum mass	70,000 Da
m/z error tolerance of spectral peaks	0.02 m/z
MS1 signal-to-noise ratio	3
MS/MS signal-to-noise ratio	1
Use MSDeconv score	False
ECScore cutoff	0.5
Number of MS1 scans to detect a feature	3
Use noise levels in single scans to filter	False
Disable final filtering of envelopes	False
Disable additional feature search	False

Table S2: Parameter settings of TopPIC for “Precursor intensity and spectral identification”

Input Parameter	Value
Fixed modification	Carbamidomethylation on cysteine
Allowed N-terminal forms	None, NME, NME_ACETYLATION, M_ACETYLATION
Maximum number of mass shift	1
Minimum value of a mass shift	-500 Da
Maximum value of a mass shift	500 Da
Use a shuffled decoy database	True
Mass error tolerance	10 ppm
Proteoform error tolerance	1.2 Da
Spectrum level cutoff type	FDR
Spectrum level cutoff value	0.01
Proteoform level cutoff type	FDR
Proteoform level cutoff value	0.01
Use TopFD Features	True

Table S3: Parameter settings of TopPIC for “Evaluation on pseudo-multiplexed MS/MS spectra”

Input Parameter	Value
Fixed modification	Carbamidomethylation on cysteine
Allowed N-terminal forms	None, NME, NME_ACETYLATION, M_ACETYLATION
Maximum number of mass shift	1
Minimum value of a mass shift	-500 Da
Maximum value of a mass shift	500 Da
Use a shuffled decoy database	False
Mass error tolerance	10 ppm
Proteoform error tolerance	1.2 Da
Spectrum level cutoff type	E-value
Spectrum level cutoff value	0.01
Proteoform level cutoff type	E-value
Proteoform level cutoff value	0.01
Use TopFD Features	True

Table S4: Parameter settings of TopMPI for evaluating δ and γ

Input Parameter	Value
Fixed modification	Carbamidomethylation on cysteine
Allowed N-terminal forms	None, NME, NME_ACETYLATION, M_ACETYLATION
Maximum number of mass shift	1
Minimum value of a mass shift	-500 Da
Maximum value of a mass shift	500 Da
Use a shuffled decoy database	True
Mass error tolerance	10 ppm
Proteoform error tolerance	1.2 Da
TopPIC spectrum level cutoff type	E-value
TopPIC spectrum level cutoff value	10,000
TopMPI spectrum level cutoff type	FDR
TopMPI spectrum level cutoff value	0.01
TopMPI use TopFD Features	Yes
TopMPI proteoform level cutoff type	FDR
TopMPI spectrum level cutoff type	0.01
α	0.2
β	0.9
γ	Various settings
δ	Various settings

Table S5: Parameter settings of TopMPI for comparing with TopPIC

Input Parameter	Value
Fixed modification	Carbamidomethylation on cysteine
Allowed N-terminal forms	None, NME, NME_ACETYLATION, M_ACETYLATION
Maximum number of mass shift	1
Minimum value of a mass shift	-500 Da
Maximum value of a mass shift	500 Da
Use a shuffled decoy database	True
Mass error tolerance	10 ppm
Proteoform error tolerance	1.2 Da
TopPIC spectrum level cutoff type	E-value
TopPIC spectrum level cutoff value	10,000
TopMPI spectrum level cutoff type	FDR
TopMPI spectrum level cutoff value	0.01
TopMPI use TopFD Features	Yes
TopMPI proteoform level cutoff type	FDR
TopMPI spectrum level cutoff type	0.01
α	0.2
β	0.9
γ	4
δ	5

Figure

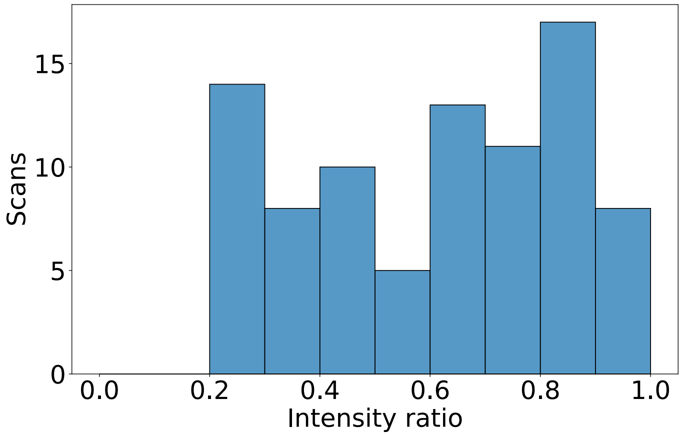

Fig. S1: The distribution of the intensity ratio of the second and first most abundant precursors in the 86 MS/MS spectra with proteoform pair identifications reported from the *E. coli* data set. The minimum value of the intensity ratio is 0.21.
